# Multiple Skin Squamous Cell Carcinomas in Junctional Epidermolysis Bullosa Due to Altered Laminin-332 Function

**DOI:** 10.3390/ijms21041426

**Published:** 2020-02-20

**Authors:** Paola Fortugno, Angelo Giuseppe Condorelli, Elena Dellambra, Liliana Guerra, Francesca Cianfarani, Lavinia Tinaburri, Vittoria Proto, Naomi De Luca, Francesca Passarelli, Francesca Ricci, Giovanna Zambruno, Daniele Castiglia

**Affiliations:** 1 Laboratory of Molecular and Cell Biology, IDI-IRCCS, via Monti di Creta 104, 00167 Rome, Italy; p.fortugno@idi.it (P.F.); e.dellambra@idi.it (E.D.); l.guerra@idi.it (L.G.); francy.cianfarani@gmail.com (F.C.); lavinia.tinaburri@gmail.com (L.T.); vitto.proto@gmail.com (V.P.); n.deluca@idi.it (N.D.L.); 2 Genetics and Rare Diseases Research Division, Bambino Gesù Children’s Hospital, IRCCS, viale di San Paolo 15, 00146 Rome, Italy; agiuseppe.condorelli@opbg.net (A.G.C.);; 3 Pathology Unit, IDI-IRCCS, via Monti di Creta 104, 00167 Rome, Italy; f.passarelli@idi.it (F.P.); francesca.ricci@idi.it (F.R.)

**Keywords:** LAMB3, disulphide bond, laminin assembly, laminin coiled-coil domain, epidermal carcinogenesis, extracellular matrix

## Abstract

Variably reduced expression of the basement membrane component laminin-332 (α3aβ3γ2) causes junctional epidermolysis bullosa generalized intermediate (JEB-GI), a skin fragility disorder with an increased susceptibility to squamous cell carcinoma (SCC) development in adulthood. Laminin-332 is highly expressed in several types of epithelial tumors and is central to signaling pathways that promote SCC tumorigenesis. However, laminin-332 mutations and expression in individuals affected by JEB-GI and suffering from recurrent SCCs have been poorly characterized. We studied a JEB-GI patient who developed over a hundred primary cutaneous SCCs. Molecular analysis combined with gene expression studies in patient skin and primary keratinocytes revealed that the patient is a functional hemizygous for the p.Cys1171* mutant allele which is transcribed in a stable mRNA encoding for a β3 chain shortened of the last two C-terminal amino acids (Cys1171-Lys1172). The lack of the Cys1171 residue involved in the C-terminal disulphide bond to γ2 chain did not prevent assembly, secretion, and proteolytic processing of the heterotrimeric molecule. Immunohistochemistry of SCC specimens revealed accumulation of mutant laminin-332 at the epithelial-stromal interface of invasive front. We conclude that the C-terminal disulphide bond is a structural element crucial for laminin-332 adhesion function in-vivo. By saving laminin-332 amount, processing, and signaling role the p.Cys1171* mutation may allow intrinsic pro-tumorigenic properties of the protein to be conveyed, thus contributing to invasiveness and recurrence of SCCs in this patient.

## 1. Introduction

Laminin-332 (LM332) is an extracellular matrix (ECM) component of the epithelial basement membrane (BM), highly abundant in the skin. It is a multidomain glycoprotein formed by three polypeptide subunits, the α3a (200 kDa), β3 (140 kDa), and γ2 (155 kDa) chains, shaped in a disulphide-bonded coiled-coil cross with one long arm and three short arms [1]. Epidermal basal keratinocytes synthesize and assemble the heterotrimeric protein within the endoplasmic reticulum (ER) where β3-γ2 heterodimers with disulphide bonds first form, followed by the alignment of the α3 chain [2]. The assembled heterotrimer is then secreted and deposited in the basal lamina in a mature form generated by proteolytic cleavage of the β3 and γ2 chains [1,2]. Outside the cells, LM332 can simultaneously bind to other ECM and cell membrane molecules, forming anchoring junctions and/or networks that are crucial for epithelial cell adhesion and wound regeneration [1,3].

Integrins α6β4 and α3β1, syndecans-1 and -4, collagens VII and XVII are important cell surface receptors and ECM ligands of laminin-332, which uses distinct subdomains of the N- and C-terminus of the coiled coil cross for these interactions: (i) the α3 laminin globules (LG1-5) for binding integrins and syndecans; (ii) laminin N-terminal (LN) and L4 subdomains and multiple laminin epidermal growth factor (EGF)-like (LE) modules to interact with the non-collagenous (NC1) domain of collagen VII and, indirectly, other laminins; and (iii) not yet identified structural motifs to interact with type XVII collagen ectodomain [3,4,5]. Distinct properties have been attributed to processed and unprocessed forms of LM332, with the former primarily implicated in epidermal–dermal cohesion of intact skin and the latter having additional functions in protein deposition, cell motility, wound healing, and squamous cell carcinoma (SCC) tumorigenesis [1,3,6,7,8]. In particular, LM332 containing unprocessed α3 LG domain is found under migrating wound keratinocytes and at the epithelial-stromal interface of SCC where its levels have been shown to correlate with tumor invasion and patient prognosis [8,9]. Besides, LM332 re-expression in an animal model of human SCC resulted as crucial for SCC tumorigenesis, and its cell and ECM interactions to integrin α6β4 and collagen VII were shown to mediate pro-tumorigenic signaling through PI3K and MAPK activation [8,10].

Loss of LM332 causes junctional epidermolysis bullosa (JEB), a spectrum of clinical phenotypes characterized by fragility of the skin and mucous membranes, blisters, and erosions that occur in response to minor frictional forces [3]. The severity of skin fragility and phenotype largely depends on the amount of LM332 produced by mutant keratinocytes, which bear recessive mutations in any of the three genes, *LAMA3A*, *LAMB3*, *LAMC2* encoding the α3a, β3, and γ2 chains, respectively. The early lethal JEB generalized severe subtype results from absent LM332, while generalized intermediate (JEB-GI) or localized JEB forms associate with variably reduced LM332 amounts. In addition, JEB-related non-blistering conditions with specific tissue manifestations result from dominant haploinsufficiency or from partial loss-of-function that spares the amount and coiled coil domain of the heterotrimer [3,11,12,13]. JEB-GI can also be due to genetic defects in *COL17A1* encoding collagen XVII [14].

Data from patient cohorts have suggested that adult JEB patients with defective LM332 are at an increased risk of developing SCC starting from their third decade of life [15]. However, the association between JEB and SCC and between LM332 mutations and propensity to tumorigenesis needs further investigation since JEB-GI is a quite rare disease subtype, patient cohorts studied so far are small and naturally occurring LM332 mutations associated with SCC development have been poorly characterized [16,17].

Here, we define the molecular basis of JEB in a patient who shows a huge propensity to develop SCCs in adult life and investigate patient tumor specimens and primary keratinocytes to gain insight into LM332 biology and JEB-related SCC tumorigenesis.

## 2. Results

### 2.1. Patient Clinical Findings

**Figure 1:**
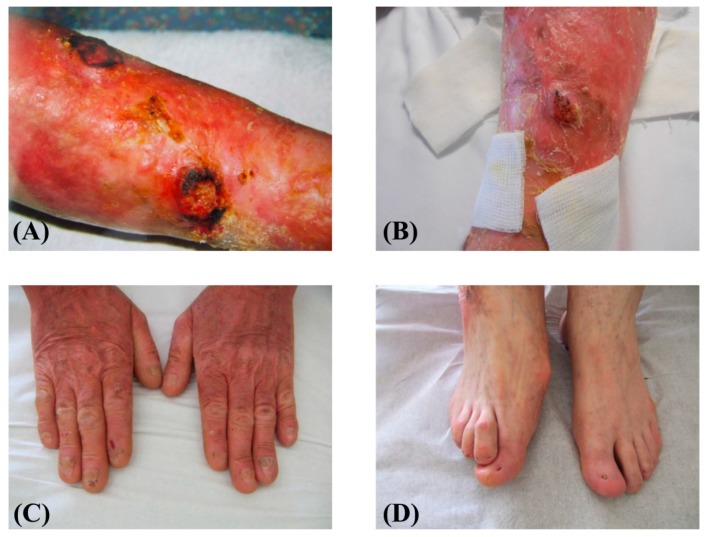
Patient clinical features. (**A**) The skin of the left leg is diffusely erythematous with several blisters, crusts, and two nodular lesions with hyperkeratotic borders, suggestive for squamous cell carcinoma (SCC), and (**B**) the right leg skin shows similar features and a single nodular lesion, also suggestive of SCC; the three nodular lesions were excised and the diagnosis confirmed by histopathology. (**C**) Skin atrophy, dyspigmentation and a few small crusts of the digits and hand dorsum. The fingernails are markedly dystrophic or absent; (**D**) absence of toenails.

A 61-year-old patient with a previous diagnosis of JEB-GI was addressed to our laboratory for molecular diagnosis. The patient had been followed in the Plastic Surgery Division of IDI-IRCCS hospital for multiple SCCs since the age of 48. He was the first child of healthy non-consanguineous parents and presented generalized skin and oral blistering since birth. Nail dystrophies developed in early infancy and led to toenail loss around age 1 year and to loss of several fingernails in childhood. Progressive scalp alopecia and loss of body, inguinal, and axillary hair occurred in adulthood. In addition, teeth showed enamel pitting which resulted in progressive tooth loss in adulthood. Skin blistering improved starting from adolescence, except on lower limbs that continued to show major skin fragility, leading to the development of unremitting chronic wounds in the fourth decade of life. At age 48, the patient underwent surgical excision of the first SCC of the lower left leg. Since then, he developed more than 90 SCCs of the left leg and about 10 SCCs of the right leg (mostly infiltrating SCCs and some in-situ carcinomas). The incidence of tumor appearance was, on average, 5 new lesions per year, with a peak of 14 carcinomas in one year. They were all histopathologically classified, according to WHO Classification of Skin Tumors [18], as well- and moderately-differentiated SCCs and, not infrequently, an additional in-situ carcinoma was observed at the border of the infiltrating carcinoma. The patient was otherwise in good general health. Physical examination showed generalized skin atrophy with a few blisters and crusts over the trunk and upper extremities. The knees and legs presented confluent erythematous crusty and hyperkeratotic lesions, chronic ulcerations and rare blisters (Figure 1A). Three nodular lesions of about 3 cm in diameter, two localized on the left leg (Figure 1A), and the third one on the right leg (Figure 1B) were also observed. Toenails and several fingernails were absent (Figure 1C,D).

### 2.2. Mutation Identification and Laminin-332 (LM332) mRNA and Protein Expression in Patient Primary Keratinocytes and Skin

**Figure 2:**
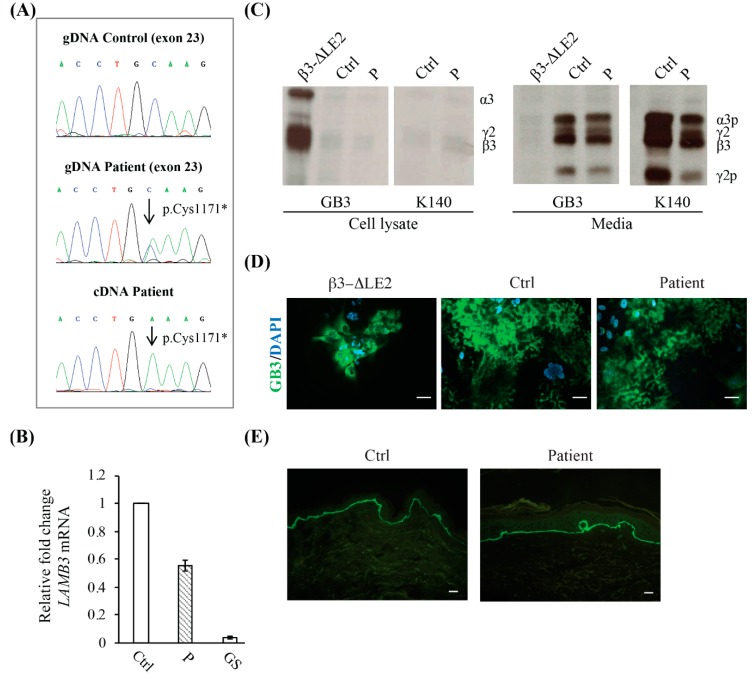
Molecular characteristics of the patient. (**A**) LAMB3 exon 23 sequence chromatogram of patient genomic DNA (gDNA) shows the p.Cys1171* compound heterozygous mutation (middle panel). A sequence chromatogram from a normal control is shown for comparison (upper panel). The sequence of a complementary DNA (cDNA) fragment reverse-transcribed from the total RNA of patient keratinocytes reveals the p.Cys1171* mutation as unique peak, indicating that the mRNA carrying this variant is stable and contributes alone to protein synthesis (lower panel). (**B**) Real-time RT-PCR shows that LAMB3 total RNA levels in patient keratinocytes (P) are reduced to almost 50% of a normal control (Ctrl), while they are dramatically decreased in a junctional epidermolysis bullosa generalized severe keratinocyte strain (GS) carrying premature termination codon mutations in both LAMB3 alleles. (**C**) Immunoprecipitation of laminin-332 (LM332) from cell lysates and media of 35S-labelled keratinocytes from our patient (P), a previously reported junctional epidermolysis bullosa generalized intermediate (JEB-GI) individual (β3-ΔLE2) with a mutation causing an unfolded β3 short arm submodule, and a normal control (Ctrl). Equal amounts of protein-bound radioactivity were immunoprecipitated with mAbs GB3 and K140 and loaded on the gels under reducing conditions. Increased levels of unprocessed LM332 are only detected in cell lysates from the β3-ΔLE2 individual (using GB3), while, in comparison, samples from our patient and normal control show barely detectable levels of the unprocessed protein (using GB3 and K140) (left panel). Conversely, the mature LM332 is barely detected in β3-ΔLE2 spent medium and highly abundant in the media from the normal control and patient keratinocytes. The levels of processed LM332 were approximately 60% in comparison to normal cells. (**D**) Immunofluorescence localization of LM332 in junctional epidermolysis bullosa (JEB) keratinocytes. The GB3 antibody (against heterotrimeric LM332) stains the protein extracellularly in the patient and control keratinocytes (middle and right panel). In contrast, staining was almost exclusively confined within the cell cytoplasm in the β3-ΔLE2 cells (left panel). Scale bars = 10 µm. (**E**) Immunofluorescence of patient skin (right panel) using GB3 antibody shows a positive staining almost comparable to control skin (left panels). Scale bars = 20 µm.

**Figure A1:**
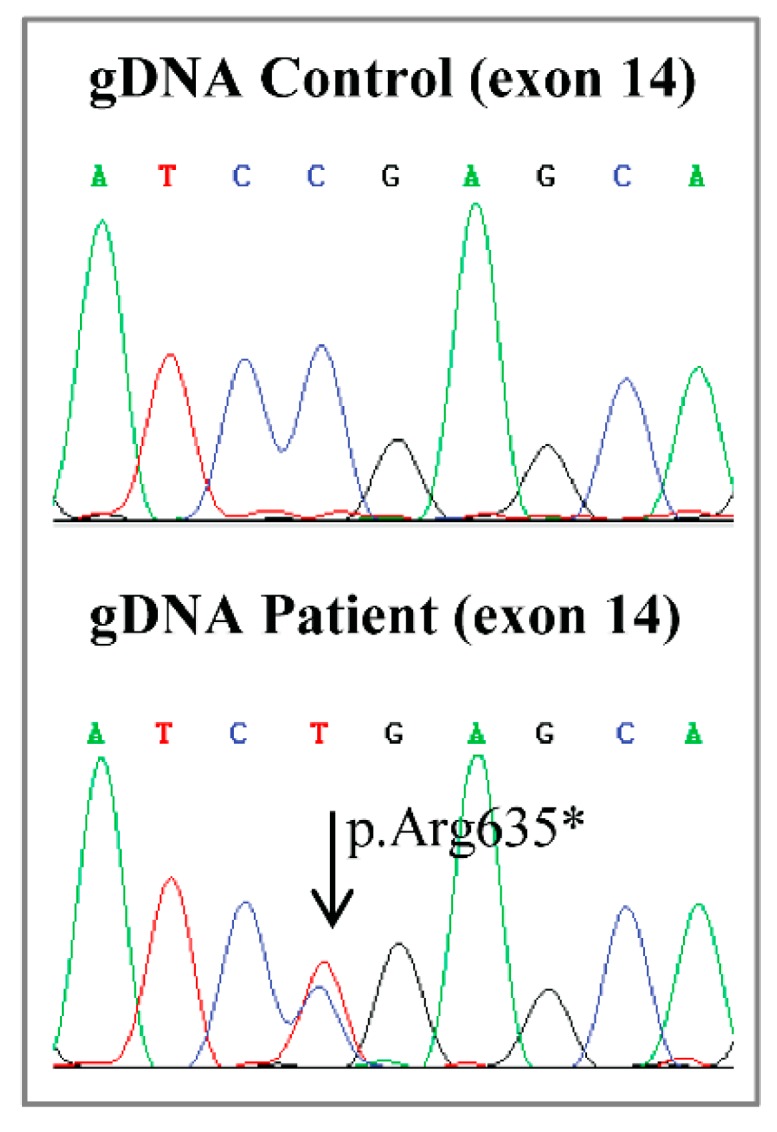
Sequence analysis of *LAMB3* exon 14 identifies the heterozygous p.Arg635* null mutation. Panels show the electropherograms of LAMB3 exon 14 sequence obtained from the genomic DNA (gDNA) of a normal control (upper) as compared to the sequence obtained from the gDNA of the patient (lower), who harbors the heterozygous C-to-T transition leading to the p.Arg635* nonsense mutation (arrow).

Sanger sequencing of the JEB-GI genes identified two heterozygous mutations in LAMB3: c.1903C>T (exon 14) (Figure A1) and c.3513C>A (exon 23) (NCBI GenBank NM_000228) (Figure 2A). The former variation is recurrent in European JEB individuals and results in p.Arg635* premature termination leading to mRNA decay [3]. The latter variant results in the p.Cys1171* change of the penultimate codon of the β3 chain. This mutation is not reported in the literature, but it is annotated in the gnomAD exome variant database at the heterozygous state (2 alleles out of 246,146, MAF: < 0.01). At mRNA level this mutation has likely no impact on mRNA stability being located in the last LAMB3 exon. Indeed, real-time reverse transcriptase-PCR of total RNA extracted from patient cultured keratinocytes revealed LAMB3 mRNA levels reduced by about 50% in comparison to normal cells (Figure 2B). In addition, direct sequencing of a cDNA fragment spanning the c.3513C>A mutation site showed that all LAMB3 transcripts produced by the cells carried this variant, thus demonstrating that the nonsense mRNA decay mechanism targeted the 50% of transcripts linked to the p.Arg635* while sparing the other 50% linked to the p.Cys1171* mutation (Figure 2A, lower panel). In agreement with mRNA levels, immunoprecipitation (IP) and SDS-PAGE analysis of cultured keratinocyte lysate using the same mAbs did not detect accumulation of mutant LM332 (precursor chains: 200 kDa for α3, 155 kDa for γ2, and 140 kDa for β3) within patient cells while protein cellular accumulation was evident in keratinocytes of a patient with a previously characterized mutation causing unfolding of the LE motif 2 of the β3 short arm (β3-ΔLE2) (Figure 2C) [19]. Consistently, spent culture medium of cultured patient cells showed bands for mature, physiologically cleaved, LM332 (165 kDa for α3 and 105 kDa for γ2), the intensity of which was estimated to be about 60% of control keratinocytes (Figure 2C). Immunofluorescence (IF) analysis of patient keratinocytes revealed a staining intensity for secreted heterotrimeric LM332 (GB3 staining) substantially similar to control cells without evidence of intracytoplasmic accumulation, which, in contrast, marked the β3-ΔLE2 cells (Figure 2D). Finally, IF examination of a perilesional skin biopsy with mAb GB3 showed a positive signal, which appeared comparable to control skin (Figure 2E). Taken together, these findings demonstrate that the patient is a functional hemizygous for the c.3513C>A: p.Cys1171* nonsense mutation. This change eliminates the last two amino acids of the β3 subunit (Cys1171-Lys1172), one of which is the cysteine residue involved in the disulphide bond between the β3 and γ2 chains at the C-terminus [1]. Lack of this bond does not influence LM332 chain assembly and secretion, as no intracytoplasmic protein retention was detected in patient skin and cultured keratinocytes.

### 2.3. LM332 Expression in Patient Squamous Cell Carcinomas (SCCs)

**Figure 3:**
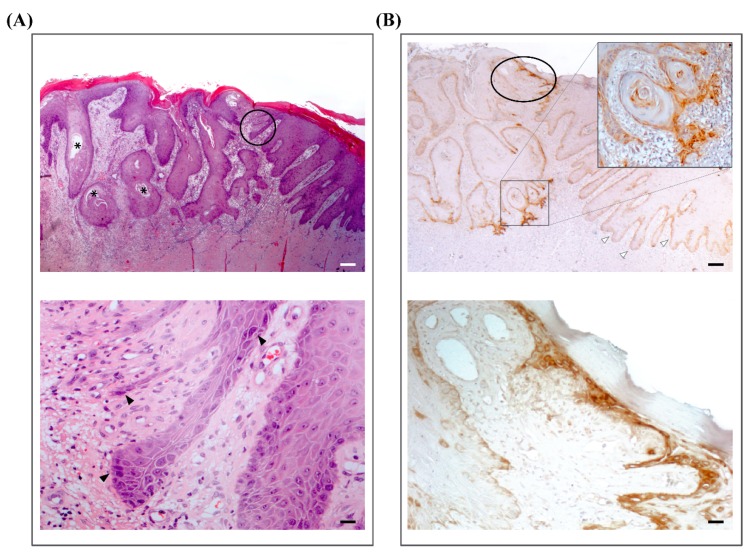
Laminin-332 expression in patient squamous cell carcinomas (SCCs). (**A**) Upper panel. Histopatological characteristics of a representative cutaneous SCC of the patient. Hematoxylin-eosin staining shows nests of squamous epithelial cells which arise from the epidermis and extend into the dermis for a variable distance. The cells have abundant eosinophilic cytoplasm and a large, often vescicular, nucleus. There is variable central keratinization and horn pearl formation, defining a well-differentiated SCC (asterisks). Next to the SCC, non-tumoral hyperproliferative epidermis is easily recognized at the right margin. Between infiltrating tumor strands and non-tumoral skin, an in-situ carcinoma is evident (black circle). Scale bar = 50 µm. Lower panel. Higher magnification of the in-situ carcinoma showing filiform strands of squamous atypical cells that are enlarged and pleomorphic with hyperchromatic nuclei (black arrowheads). These cells replace basal cells and show loss of polarity. Non-tumoral hyperproliferative epidermis is present at the right margin. Scale bar = 6 µm. (**B**) Upper panel. Immunohistochemistry for LM332 in a serial section of the SCC shown in panel (A) using an anti-laminin β3 chain antibody (clone 17, Kalinin B1, BD Transduction Laboratories, Lexington, KY, USA). Black circle indicates the area of the in-situ carcinoma (enlarged in lower panel). Staining is strongly positive in areas of invasive SCC, in particular at the tumor leading edge (inset: higher magnification image). Note that hyperproliferative non-tumoral skin at the right margin shows a predominantly linear deposit along the cutaneous basement membrane zone (white arrowheads). Scale bar = 50 µm. Lower panel. LM332 accumulation within the area of the in-situ carcinoma. Scale bar = 6 µm.

Since LM332 is highly expressed in cutaneous SCCs from the general population [9] and its expression correlates with tumor invasiveness, the presence of LM332 was also investigated by immunohistochemistry on formalin-fixed, paraffin-embedded (FFPE) sections of patient SCCs using an anti-β3 chain antibody. All locally invasive and in-situ SCCs evaluated (*n* = 10) (Figure 3A,B) showed accumulation of LM332, in particular at the invasive edge of the tumors (Figure 3B).

## 3. Discussion

**Figure 4:**
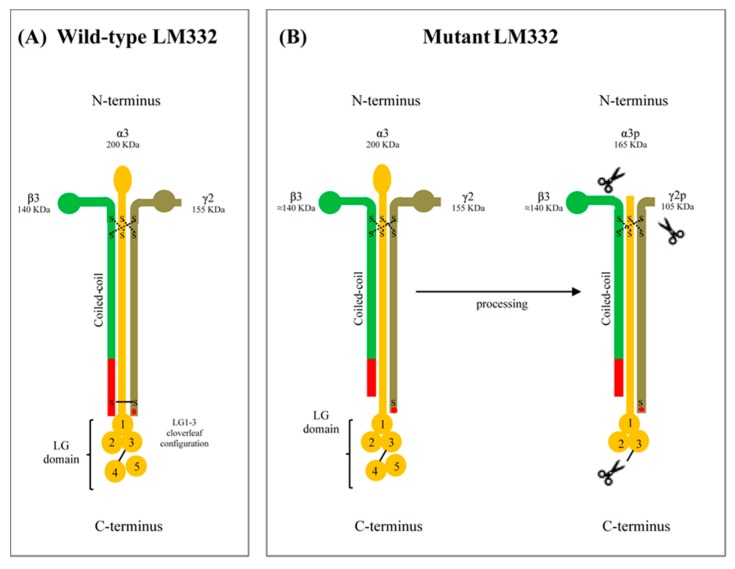
Schematic of the laminin-332 (LM332) structure and its maturation process. (**A**) Unprocessed, intracellular form of the wild-type LM332. It consists of three chains: α3 (200 kDa), β3 (140 kDa) and γ2 (155 kDa). These chains fold together in a coiled coil cross. The heterotrimeric molecule is stabilized by disulphide bonds at the N-terminus (dashed lines) and C-terminus. The C-terminal bond connects Cys1171 in the β3 chain to Cys1184 in the γ2 chain (solid line). (**B**) Left panel. Unprocessed, intracellular form of the mutant LM332 of our patient. Mutation p.Cys1171* in the β3 chain results in absence of the disulphide bond at the C-terminus. (**B**) Right panel. Processed, extracellular form of the mutant LM332. Similar to the wild-type form, the mutant LM332 is normally secreted and proteolytically cleaved within the α3 and γ2 chains (cleavage sites are indicated by scissors), and then deposited in the extracellular matrix. The maturation process of LM332 outside the cells determines the reduction of α3 chain size from 200 to 165 kDa (α3p), and of γ2 chain from 145 kDa to 105 kDa (γ2p). The last 20 C-terminal residues of the β3 chain (red trait) and Glu1191 in the γ2 (red dot) that are important structural determinants for integrin recognition are indicated.

Studies in individuals carrying naturally occurring mutations add and improve knowledge on LM332 biology and biophysical chemistry. Our patient can be considered a functional hemizygous for the LAMB3 p.Cys1171* mutation at mRNA and protein level, thus providing evidence that qualitative differences in LM332 affect dermal–epidermal adhesion and lead to JEB. The functional hemizygosity, which results from the combination of the p.Cys1171* with the p.Arg635* null variant on the other allele, evokes the genetic condition of a heterozygous carrier for a null mutation, who expresses half dose of normal mRNA and protein and does not suffer from skin blistering [20]. Therefore, the p.Cys1171* mutation, which does not affect LAMB3 gene expression, results in a functional LM332 defect responsible for the skin fragility observed in the patient. Moreover, this mutation highlights the role of the C-terminal disulphide bond (between Cys1171 in the β3 chain and Cys1184 in the γ2 chain) in stabilizing assembled LM332 heterotrimers (Figure 4).

Indeed, chain specificity and assembly by coiled-coil formation in LM332 can proceed without the C-terminal disulphide bridge, since interchain non-covalent hydrophobic and ionic interactions are the only driving force of protein folding [21,22,23]. This matches with our findings in patient skin and keratinocytes showing that the mutant LM332 is correctly assembled, secreted, and processed without evidence of a defective folding (Figure 4). We inferred that the absence of disulphide bond may influence the LM332 interaction network outside the cell. Indeed, in vitro biophysical analyses demonstrated that the C-terminal disulphide bridge between recombinant β and γ fragments allows laminin β-γ dimer assembly to be more selective, efficient, and less dependent from the concentration of each chain subunit [24]. The β-γ disulphide bridge confers thermodynamic stability to the assembled chains, a status that most likely provides LM332 long arm with the conformational flexibility needed to recognize and simultaneously/dynamically interact with its binding partners after secretion and deposition in the ECM [3,24]. Moreover, the C-terminal tail of β chains contributes to the integrin binding affinity of laminins thanks to its proximity to the cloverleaf configuration of LG1-3 subdomain, where integrin binding sites are placed, and because of the disulphide bridge to the γ chain near the C-terminus, where the glutamic acid residue (Glu1191 in γ2) critical for integrin recognition is situated (Figure 4) [1,25]. Thus, it is conceivable that mutation p.Cys1171* in the β3 chain can lessen the adhesive interactions between LM332 and integrins. On the other hand, the interactions of LM332 with its binding partners promote epidermal carcinogenesis through signaling that is uncoupled from stable adhesion [10,26]. These non-adhesive extracellular cues are theoretically preserved in patient skin, since the mutant LM332 produced by our patient has normal laminin β3 LE domains in the short arm to bind collagen VII NC1 domain, and normal LG subdomains to recognize integrin and syndecans receptors [8,9,26,27].

Here, we show that the mutant LM332 is correctly secreted and proteolytically processed in primary keratinocytes and expressed in patient skin in amounts comparable to control skin. Its expression pattern in patient tumors is similar to that observed in SCCs from non-JEB individuals, with accumulation observed at the invasive edges of tumors [9,10,26].

Since the lack of LM332 disrupts SCC tumorigenesis of HRAS/IkBα-transformed human epidermis grafted in immunodeficient mice, while cell adhesion substrates and integrin-mediated signaling support tumor growth and invasion [7,8], we assume that specific LM332 mutations that spare protein amounts and signaling functions may allow intrinsic pro-tumorigenic properties of the protein to be conveyed. Indeed, it is remarkable that, in addition to the patient here described, another JEB-GI patient affected by a homozygous LAMA3A mutation resulting in unusually high amounts (up to 50%) of processed LM332 in the skin, and cultured keratinocytes also showed a high propensity to develop SCC in adulthood (20 tumors by age 62) [17]. The number of SCCs developed by each of these two patients is surprisingly higher compared to that found in literature (2 SCCs per patient, as median), notably in the absence of any tendency to metastasize (Table 1) [15,16].

In parallel to the aberrant signaling, skin fragility consequent to defective adhesion could help SCC tumorigenesis. Repeated mechanical traumas in EB lead to chronic wounds accompanied by tissue inflammation, subsequent ECM remodeling/dermal fibrosis, and skin microenvironment alterations that form the ground for SCC development and recurrence [31,32]. Of note, all SCCs occurred in lower extremities mostly in the pretibial region and within areas of chronic blistering, long-standing erosions/ulcers, or atrophic scarring. Interestingly, the histopathology of surgically excised tumors often evidenced at the border of the infiltrating SCC also the presence of an in-situ carcinoma, indicating that new tumors arise in the field surrounding the preceding SCCs (Figure 3). This finding further points to the role of the skin microenvironment in JEB SCC tumorigenesis.

## 4. Materials and Methods

### 4.1. Patient Samples, Immunofluorescence, Molecular Analysis

Skin biopsies and a blood sample were obtained after patient’s informed consent, with the approval of the IDI-IRCCS Ethics Committee and in conformity with the Helsinki guidelines. Immunofluorescence analysis of a patient’s skin biopsy and primary keratinocyte cultures from a second biopsy were performed as described [33]. Primary keratinocytes grown on glass coverslips in 24-well tissue culture plates were subjected to an indirect immunofluorescence procedure. In brief, cells were fixed 20 min at room temperature (r.t.) in PBS containing calcium chloride and magnesium chloride (PBS+), and 3% formaldehyde. Cells were then rinsed three times in PBS+, and then permeabilized 2 min at r.t. with PBS+ 0,1% Triton X-100 and washed with PBS+ before exposure to antibodies [34,35]. Genomic DNA extracted from blood was used to generate and sequence PCR fragments of the entire coding region and splice site junctions of *LAMB3*, *LAMC2*, *LAMA3*, and *COL17A1* as described [36,37]. The identified mutations were numbered according to the coding region (ATG as codon 1) of the *LAMB3* transcript (NM_000228.3).

### 4.2. LAMB3 mRNA Expression and LM332 Protein Analysis

Total RNA was purified from patient and control keratinocytes and used to assess *LAMB3* mRNA expression level by Real-Time PCR. Amplification was performed by using SYBR-Green Master Mix (Applied Biosystems, Foster City, CA, USA). Primer pairs used are described in Ref. [19]. Glyceraldehyde-3-phosphate dehydrogenase (GAPDH) served as housekeeping gene.

A complementary DNA (cDNA) fragment across mutation c.3513C>A was generated by RT-PCR and then sequenced in both orientations with the following primers: 5′-GTTGGGTCAGAGTTCCATGC-3′ (forward, exon 22) and 5′-TGAAAGTCTCCTGGAGATGG-3′ (reverse, exon 23).

Radioimmunoprecipitation of cell lysate and culture medium was performed as described, using K140 (mouse mAb to the laminin β3 chain), and GB3 (mouse mAb which recognizes the LM332 heterotrimer) [33]. Immunoprecipitated proteins were analyzed by SDS-PAGE on 6% polyacrylamide gels under reducing conditions, followed by autoradiography. Quantification of autoradiograms was performed by densitometric scanning with a Gel Doc 1000 (Bio-Rad, Hercules, CA, USA).

### 4.3. Immunohistochemistry

FFPE sections (3μm from surgically excised SCCs) were stained with hematoxylin and eosin and processed for immunohistochemistry. Antigen retrieval was performed by incubation at 98 °C for 15 min in citrate-EDTA buffer, pH 9.0. The sections were then treated with 4% BSA and 5% horse serum for 1 hr at r.t. The anti-LM332 immunoreactivity was obtained with the anti-laminin β3 chain monoclonal antibody (clone 17, Kalinin B1, BD Transduction Laboratories, Lexington, KY, USA) (dilution 1:100). Slides were then washed with PBS (3 times for 5 min) and treated with 3% H_2_O_2_ for 30 min. A biotinylated anti-mouse secondary antibody (Vector Laboratories, Burlingame, CA, USA; 1:150 dilution) was applied for 1h at r.t., followed by ABC reagent (Avidin and Biotinylated horseradish peroxidase macromolecular Complex–Vectastain Elite ABC kit; Vector Laboratories). Diaminobenzidine–tetrahydrochloride (DAB, DAKO Corporation, Carpinteria, CA, USA) and hematoxylin served for staining and counterstaining, respectively. The uninvolved skin within each section provided the positive control.

## 5. Conclusions

The present study identified a naturally occurring LM332 mutation in a JEB-GI individual who has developed, in his adult life, over a hundred primary cutaneous SCCs. Our findings demonstrate that the mutation affects a structural determinant of LM332 stability (the C-terminal disulphide bridge) previously unrecognized in JEB pathology. Due to the intrinsic pro-tumorigenic proprieties of LM332, a subset of JEB patient with qualitative, rather than quantitative, LM332 defects may have a much higher risk than described in JEB (median of two SCC/patient) to develop multiple and recurrent cutaneous SCCs requiring strict follow-up.

## Figures and Tables

**Table 1:** Reported cases of squamous cell carcinoma in molecularly characterized JEB-GI patients.

Patient	Sex	Mutation (Protein Designation)	Expression of Mutant Protein (Skin IF)	Age (y) at First SCC	N° of SCCs	Localization	Differentiation in Histopathology	Metastasis	Ref.
**1**	M	*LAMB3* p.Arg635*+p.Cys1171*	LM332 normal	48	100	Lower extremities	Well, moderately	No	This study
**2**	M	*LAMA3* p.Leu1648Trpfs*32 (h)	LM332 slightly reduced	42	>20	Lower extremities	Well, moderately	No	[17]
**3**	F	*COL17A1* p.Ser1079Cysfs*26 (h)	COLXVII negative	42	4	Lower extremity	Well, moderately, poorly	Yes	[15]
**4**	M	*LAMB3* p.Glu210Lys (h)	LM332 reduced	48	2	Lower extremity	Well, moderately	No	[15]
**5**	F	*LAMB3* p.Leu11Profs*43+p.Glu210Lys	LM332 reduced	61	2	Lower extremity	Well	No	[15]
**6**	M	*LAMB3* p.Glu210Lys+p.Arg635*	LM332 reduced	28	9	Lower extremity	Well, poorly	Yes	[15]
**7**	M	*LAMB3* p.Leu11Profs*43+p.Gln834*	LM332 negative	39	>8	Lower extremities	Well	No	[28]
**8**	M	*LAMB3* p.Leu11Profs*43+p.Gln834*	LM332 negative	32	>4	Lower extremities	Well	Yes	[28]
**9**	F	*LAMB3* p.Arg635*+p.Thr350Pro	LM332 reduced	70	>6	Sacrum, buttock	Well, moderately	No	[29]
**10**	M	*COL17A1* p.Ser1300Cysfs*29 (h)	COLXVII negative	58	1	Lower extremity	Well	No	[30]

Abbreviations: IF, immunofluorescence; y, years; SCC, squamous cell carcinoma, M, male; F, female; LM332, laminin-332; h, homozygous.

## Abbreviations

JEB	Junctional epidermolysis bullosa
JEB-GI	Junctional epidermolysis bullosa, generalized intermediate subtype
SCC	Squamous cell carcinoma
LM332	Laminin-332
ECM	Extracellular matrix
